# Nonsteroidal anti-inflammatory drugs and breast cancer risk in the National Institutes of Health–AARP Diet and Health Study

**DOI:** 10.1186/bcr2089

**Published:** 2008-04-30

**Authors:** Gretchen L Gierach, James V Lacey, Arthur Schatzkin, Michael F Leitzmann, Douglas Richesson, Albert R Hollenbeck, Louise A Brinton

**Affiliations:** 1 Hormonal and Reproductive Epidemiology Branch, Division of Cancer Epidemiology and Genetics, National Cancer Institute, National Institutes of Health, 6120 Executive Blvd, Suite 550, Rockville, MD 20852-7234, USA; 2 Cancer Prevention Fellowship Program, Office of Preventive Oncology, National Cancer Institute, National Institutes of Health, 6120 Executive Blvd, Suite T-41, Bethesda, MD 20892-7361, USA; 3 Nutritional Epidemiology Branch, Division of Cancer Epidemiology and Genetics, National Cancer Institute, National Institutes of Health, 6120 Executive Blvd, Suite 320, Rockville, MD 20852-7232, USA; 4 Organizational and Tracking Research Department, AARP, 601 E Street NW, Washington, DC 20049, USA

## Abstract

**Introduction:**

By inhibiting cyclooxygenase-2, nonsteroidal anti-inflammatory drugs (NSAIDs) decrease aromatase activity and might reduce breast cancer risk by suppressing estrogen synthesis. Epidemiologic evidence for a protective role of NSAIDs in breast cancer, however, is equivocal.

**Methods:**

We tested NSAID use for its association with breast cancer incidence in the National Institutes of Health–AARP Diet and Health Study, where 127,383 female AARP (formerly known as the American Association of Retired Persons) members with no history of cancer, aged 51 to 72 years, completed a mailed questionnaire (1996 to 1997). We estimated relative risks of breast cancer for NSAID exposures using multivariate Cox proportional hazards regression models. The state cancer registry and mortality index linkage identified 4,501 primary incident breast cancers through 31 December 2003, including 1,439 estrogen receptor (ER)-positive cancers and 280 ER-negative cancers.

**Results:**

Proportional hazards models revealed no statistically significant association between overall NSAIDs and total breast cancer. As cyclooxygenase inhibition by aspirin (but not other NSAIDs) is irreversible, we tested associations by NSAID type. Although we observed no significant differences in risk for daily use (versus nonuse) of aspirin (relative risk = 0.93, 95% confidence interval = 0.85 to 1.01) or nonaspirin NSAIDS (relative risk = 0.96, 95% confidence interval = 0.87 to 1.05), risk of ER-positive breast cancer was significantly reduced with daily aspirin use (relative risk = 0.84, 95% confidence interval = 0.71 to 0.98) – a relationship not observed for nonaspirin NSAIDS. Neither aspirin nor nonaspirin NSAIDs were associated with risk of ER-negative breast cancer.

**Conclusion:**

Breast cancer risk was not significantly associated with NSAID use, but daily aspirin use was associated with a modest reduction in ER-positive breast cancer. Our results provide support for further evaluating relationships by NSAID type and breast cancer subtype.

## Introduction

Numerous epidemiologic studies have examined the association between nonsteroidal anti-inflammatory drugs (NSAIDs) and breast cancer, with equivocal results. Most, but not all, case–control studies have found relative risk reductions from 20% to 40% [1-3] (reviewed in [4-7]). Results from prospective studies have been less consistent, with eight reports finding no association [8-15], two studies observing an increased risk [16,17], one study reporting a U-shaped association [18], and six studies demonstrating a reduced risk [19-24]. In the Women's Health Study randomized clinical trial, alternate-day use of low-dose aspirin for an average of 10 years did not reduce the incidence of breast cancer [25]. Given the potential public health impact of NSAIDs as chemopreventive agents for breast cancer, an increased understanding of the relationship between NSAIDs and breast cancer is needed.

A protective role of NSAIDs in breast carcinogenesis is biologically plausible. *In vitro *and animal studies have consistently shown that NSAIDs inhibit cyclooxygenase (COX)-1 and COX-2, which cyclize and oxygenate arachidonic acid, eventually producing prostaglandins [26]. While inhibiting COX-1 reduces both platelet aggregation and gastric cytoprotection [27], inactivating COX-2 may interrupt the carcinogenic process in the breast via multiple pathways – including inhibition of angiogenesis, promotion of apoptosis, and suppression of estrogen synthesis via decreased aromatase activity [28].

Since aspirin and nonaspirin NSAIDs have different biologic effects (aspirin permanently inactivates COX-2 [29]), separate evaluation is needed with respect to breast cancer risk. Interpretation of prior epidemiologic studies is complicated by incomplete attention to NSAID type [5]. Furthermore, the studies that have examined the effect of NSAIDs on breast cancer risk by tumor characteristics, such as hormone receptor (HR) status [3,13,16-18,23,24,30,31] and stage at diagnosis [21,30,32], also present conflicting results. The relatively few studies that have tested for effect modification by participant characteristics (for example, body mass index, menopausal hormone therapy use, or smoking status) have produced inconclusive findings [13,17,18,20,22,24,25,32,33]. The large cohort of women participating in the prospective National Institutes of Health (NIH)–AARP Diet and Health Study allowed us to evaluate these questions.

## Materials and methods

### Study population

The NIH–AARP Diet and Health Study design and methodology have been described in detail [34]. Briefly, the NIH–AARP Study is a prospective cohort study of diet, health-related behaviors and cancer.

The study was initiated in 1995 and 1996 when a questionnaire was mailed to 3.5 million members of the AARP (formerly known as the American Association of Retired Persons) aged 50 to 71 years, who resided in one of six states (California, Florida, Pennsylvania, New Jersey, North Carolina, and Louisiana) or two metropolitan areas (Atlanta, Georgia and Detroit, Michigan) that have large AARP membership, large minority populations, and high-quality cancer registries. The questionnaire captured diet history, demographic characteristics, reproductive history, menopausal status, and family history of cancer. Of the 617,119 men and women (17.6%) who returned the questionnaire, 567,169 satisfactorily completed it.

In 1996 and 1997 a second questionnaire was sent to collect additional information on family history of cancer, anthropometry, and use of menopausal hormone therapy (HT) and other medications, including NSAIDs. A total of 337,074 men and women completed this questionnaire.

After excluding individuals who died or moved out of the cancer registry ascertainment area before their second questionnaire was received and scanned (*n* = 2,166), excluding proxy respondents to the baseline (*n* = 6,959) or second (*n* = 3,424) questionnaires, and excluding 188,117 men, the study population included 136,408 potentially eligible women

The study was approved by the Special Studies Institutional Review Board of the US National Cancer Institute.

### Assessment of nonsteroidal anti-inflammatory drug exposure and covariates

The second questionnaire asked whether aspirin products (generic aspirin, Bayer, Bufferin, Anacin, Ecotrin, or Excedrin) and nonaspirin NSAIDs (generic ibuprofen, Advil, Nuprin, Motrin, Naprosyn, and so on) had been used in the past 12 months. As acetaminophen is an analgesic with weak anti-inflammatory activity [35], participants were instructed not to include 'Tylenol, acetaminophen, or any other pain relievers' in nonaspirin NSAIDs. Aspirin or nonaspirin NSAID users were asked to indicate their frequency of usual use: fewer than 2 times per month, 2 to 3 times per month, 1 to 2 times per week, 3 to 4 times per week, 5 to 6 times per week, 1 time per day, or 2 or more times per day – which we collapsed to categories of nonuse, <1/week, 1 to 6/week, and ≥1/day. The dose, duration, and indication for use were not collected. In the current report, 'any NSAID' combines the use of aspirin and nonaspirin NSAIDs.

While the demographic characteristics, lifestyle, and reproductive history were largely obtained through the first questionnaire, the second questionnaire ascertained a self-reported history of hypertension, mammogram screening, and vigorous physical activity. Menopausal hormone therapy use and history of breast cancer in a first-degree relative were derived from information collected on both the first and second questionnaires.

### Cohort follow-up

Cohort members were followed annually for mailing address changes and vital status. Address changes were identified through linkage to the US Postal Service's National Change of Address database, through US Postal Service updates received with undeliverable mail, through use of other address change update services, and through participants' notifications. Vital status was updated through linkage to the Social Security Administration Death Master File and was verified by the National Death Index.

### Ascertainment of breast cancer

Incident *in situ *breast cancers and invasive breast cancers were initially identified through probabilistic linkage to eight state cancer registries using first and last name, address, sex, date of birth, and Social Security Number from the baseline questionnaire. The cancer registry ascertainment area was recently expanded to include three additional states (Texas, Arizona, and Nevada) to capture cancer occurring among participants who moved to those states during follow-up. Each registry has been certified by the North American Association of Central Cancer Registries for meeting the highest standards of data quality.

Breast cancer estrogen receptor (ER) status was coded as described in the American Joint Committee on Cancer's Collaborative Staging Site-Specific Factors Manual, with a threshold of >10 fmol cytosol protein per milligram for a positive tumor; however, the ER status was not reported by the Florida, Michigan, and Pennsylvania cancer registries. Histology was defined using the International Classification of Diseases for Oncology codes, third edition [36]. A previous validation study in this cohort estimated that registry linkage validly identified approximately 90% of all incident cancers [37]. The date of death for fatal cancers was identified through linkage to the National Death Index.

### Analytic sample

Because information regarding NSAID use was not collected on the baseline questionnaire, we limited analyses to the 136,408 women who completed the second questionnaire. We excluded 9,022 women who reported a personal cancer history other than nonmelanoma skin cancer on either questionnaire (including 942 breast cancers), 1,259 women who were missing information on NSAID use (including 52 breast cancers), and three women with no follow-up. Thus, 126,124 women were included in the present analyses.

Through 31 December 2003, 4,501 women developed breast cancer (781 *in situ*, 3,703 invasive, and 17 missing stage). Among the 2,282 invasive breast cancer cases from states that collected the ER status, 1,439 were coded as ER-positive and 280 as ER-negative (the ER status was unavailable for the remaining 563 invasive cases). There were no substantial risk factor differences between women from states where ER information was available and states where it was unavailable. The majority of invasive breast cancers were ductal carcinomas (*n* = 2,409), followed by lobular (*n* = 400), mixed (*n* = 310), and other (*n* = 584) histologic types.

### Statistical analysis

Cox proportional hazards models were used to estimate hazard ratios and 95% confidence intervals (CIs) for breast cancer associated with NSAID use; age was the time scale [38], and ties were handled by enumeration [39]. Follow-up began at the age at which the second questionnaire was received and scanned, and continued through the earliest of the following dates: participant diagnosed with breast cancer, moved out of her registry catchment area, died from any cause, or 31 December 2003. To test the proportional hazards assumption, we generated time-dependent covariates by including interactions of each predictor with the natural log of age (the time metric); probability values for all time-dependent covariates were >0.05, consistent with hazards that are proportional.

Multivariate models were used to control for age at entry (years), race/ethnicity (white versus other/unknown), age at first birth (nulliparous, <20 years, 20 to 24 years, 25 to 29 years, ≥30 years, or unknown), HT use (never used, estrogen only, estrogen–progestin, or other/unknown), number of breast biopsies (0, 1, 2, ≥3, or unknown), alcoholic drinks per day (0, <1, 1 to 3, ≥3, or unknown), history of hypertension (no, yes, or unknown), and family history of breast cancer in a first-degree relative (no, yes, or unknown). Models examining frequency of aspirin use and breast cancer risk also included terms for frequency of nonaspirin NSAID use and *vice versa*. Tests for linear trends across the NSAID exposure categories were calculated by treating these categorical variables as ordinal variables.

In subsequent models, we adjusted for calendar time and several additional factors, including ages at menarche and menopause, mammogram in the past 3 years, self-reported heart disease, self-rated health quality, physical activity, and body mass index (BMI). The results were essentially the same and are not shown here.

We used a likelihood ratio test, comparing models with and without the interaction terms, to separately examine effect modification by age at entry (<57 years, 57 to 60 years, 61 to 64 years, 65 to 68 years, or ≥69 years), HT use (never, estrogen only, estrogen–progestin, or other/unknown), BMI (<25 kg/m^2^, 25 to 29 kg/m^2^, ≥30 kg/m^2^, or unknown), and smoking status (never, former, current, or unknown). In addition, we examined whether the relationship between NSAIDs and breast cancer incidence differed by ER status (positive or negative), by stage at diagnosis (*in situ *or invasive disease), and by histologic type (ductal, lobular, or mixed).

Probability values <0.05 were considered statistically significant. All tests of statistical significance were two-tailed. Analyses were performed using SAS software release 9.1.3 (SAS Institute Inc., Cary, NC, USA).

## Results

Among the 126,124 mostly white, postmenopausal women in this report, 65.6% reported ever-use of aspirin in the past 12 months. Among all women, 31.8% took aspirin less than once per week, 16.1% used aspirin one to six times per week, and 17.7% were daily aspirin users. Slightly fewer women reported ever-use of nonaspirin NSAIDs: 31.5% took nonaspirin NSAIDs less than once per week, 15.2% used nonaspirin NSAIDs one to six times per week, and 13.3% were daily nonaspirin NSAID users. Nearly 85% of women in our study reported any NSAID use within the past 12 months.

In general, aspirin and nonaspirin NSAID users shared similar characteristics (Tables 1 and 2). Compared with women who did not use aspirin or nonaspirin NSAIDs in the past 12 months, women who had used aspirin or nonaspirin NSAIDs on a daily basis were more likely to be white, and to reportedly drink alcohol, smoke, use HT, have fair/poor overall health, and to report a history of hypertension. Daily aspirin users were more likely to be older and to report a history of stroke and heart disease. Daily nonaspirin NSAID users were more likely to be younger, parous, and obese (BMI ≥30 kg/m^2^), and to have a history of breast biopsy.

**Table 1:** Distribution of select risk factors across categories of aspirin use among 126,124 women, National Institutes of Health–AARP Study

	Frequency of aspirin use in past 12 months							
	
	None		<weekly		1–6 times/week		1+/day	
	
Characteristic	*n*	%^a^	*n*	%^a^	*n*	%^a^	*n*	%^a^
Age at second questionnaire								
<57 years	8,178	19.0	8,629	21.7	4,029	20.1	2,936	13.3
57–60 years	8,317	19.4	8,211	20.6	4,022	20.1	3,566	16.2
61–64 years	9,750	22.7	9,025	22.7	4,644	23.2	5,132	23.2
65–68 years	11,085	25.8	9,459	23.8	4,881	24.3	6,607	29.9
69+ years	5,630	13.1	4,457	11.2	2,481	12.4	3,839	17.4
Race/ethnicity								
Caucasian/non-Hispanic white	38,339	89.2	36,340	91.4	18,561	92.5	20,381	92.3
Other/unknown	4,621	10.8	3,441	8.6	1,496	7.5	1,699	7.7
Education								
<High school/high school grad	13,537	32.5	10,675	27.5	5,769	29.6	6,857	32.0
Post-high school+	28,171	67.5	28,110	72.5	13,696	70.4	14,554	68.0
Body mass index at baseline								
<25 kg/m^2^	18,461	44.7	18,855	48.9	9,040	46.6	8,718	40.8
25–30 kg/m^2^	13,144	31.8	12,331	32.0	6,407	33.0	7,290	34.2
30+ kg/m^2^	9,719	23.5	7,342	19.1	3,965	20.4	5,335	25.0
Smoking								
Never	19,321	46.3	18,123	47.0	8,996	46.4	8,984	42.1
Former	16,758	40.2	15,314	39.7	7,735	39.9	9,184	43.0
Current	5,630	13.5	5,137	13.3	2,656	13.7	3,193	14.9
Alcoholic drinks								
0 drinks per day	14,515	33.8	9,136	23.0	4,784	23.9	7,009	31.7
<1 drink per day	23,322	54.3	24,771	62.3	12,299	61.3	12,111	54.9
1–3 drinks per day	4,037	9.4	4,727	11.9	2,382	11.9	2,297	10.4
3+ drinks per day	1,086	2.5	1,147	2.9	592	3.0	663	3.0
Self-reported general health								
Excellent/very good/good	36,189	85.6	36,638	93.3	17,869	90.4	17,868	82.4
Fair/poor	6,094	14.4	2,639	6.7	1,891	9.6	3,814	17.6
Stroke								
No	42,293	98.4	39,557	99.4	19,844	98.9	21,112	95.6
Yes	667	1.6	224	0.6	213	1.1	968	4.4
Heart disease								
No	39,820	92.7	38,292	96.3	18,924	94.4	17,561	79.5
Yes	3,140	7.3	1,489	3.7	1,133	5.6	4,519	20.5
High blood pressure								
No	24,240	60.2	25,179	67.7	11,484	61.5	9,502	46.1
Yes	16,001	39.8	11,997	32.3	7,192	38.5	11,117	53.9
Mammogram in past 3 years								
Once or less	13,226	31.0	12,161	30.8	6,043	30.4	6,496	29.8
Yes, more than once	29,408	69.0	27,298	69.2	13,827	69.6	15,327	70.2
Age at menarche								
<13 years	21,222	49.8	18,850	47.8	9,679	48.7	11,036	50.4
13–14 years	17,452	41.0	17,101	43.4	8,407	42.3	8,859	40.5
15+ years	3,909	9.2	3,492	8.9	1,792	9.0	1,981	9.1
Parity								
Nulliparous	6,253	14.8	5,996	15.3	2,808	14.2	3,089	14.3
One	4,459	10.6	4,056	10.4	2,009	10.2	2,172	10.0
Two	11,052	26.2	10,488	26.8	5,290	26.8	5,406	25.0
Three or more	20,431	48.4	18,578	47.5	9,608	48.7	10,987	50.7
Age at first live birth								
Nulliparous	6,253	14.8	5,996	15.3	2,808	14.2	3,089	14.2
<20 years	7,385	17.4	6,016	15.3	3,319	16.8	3,895	17.9
20–24 years	18,472	43.6	17,112	43.6	8,861	44.9	9,800	45.1
25–29 years	7,636	18.0	7,582	19.3	3,632	18.4	3,739	17.2
30+ years	2,575	6.1	2,499	6.4	1,129	5.7	1,191	5.5
Age at menopause								
Premenopausal	1,462	3.4	1,738	4.4	742	3.7	445	2.0
<45 years	2,834	6.6	2,429	6.1	1,266	6.3	1,593	7.2
45–49 years	6,543	15.2	6,316	15.9	2,997	14.9	3,358	15.2
50–54 years	11,362	26.4	11,870	29.8	5,415	27.0	5,732	26.0
55+ years	2,650	6.2	2,555	6.4	1,218	6.1	1,284	5.8
Surgical menopause	16,770	39.0	13,577	34.1	7,754	38.7	8,975	40.6
Postmenopausal, age unknown	1,339	3.1	1,296	3.3	665	3.3	693	3.1
Hormone therapy formulation								
Never used	17,328	42.4	15,676	41.2	7,201	37.6	8,371	39.8
Estrogen therapy only	11,901	29.1	10,157	26.7	5,844	30.5	6,724	32.0
Estrogen–progestin therapy	11,681	28.6	12,196	32.1	6,100	31.9	5,945	28.3
Moderate/vigorous physical activity during past 10 years								
Never	1,801	4.3	1,033	2.6	545	2.8	878	4.0
Rarely	4,911	11.6	4,012	10.2	1,986	10.1	2,539	11.7
<1 hour/week	4,473	10.6	4,102	10.5	2,107	10.7	2,347	10.8
1–3 hours/week	10,495	24.9	10,085	25.7	5,240	26.6	5,457	25.1
4–7 hours/week	10,379	24.6	10,438	26.6	5,311	26.9	5,371	24.7
>7 hours/week	10,100	24.0	9,548	24.3	4,546	23.0	5,122	23.6
Number breast biopsies								
None	32,240	75.8	30,183	76.6	14,985	75.4	16,551	75.8
One	6,819	16.0	6,175	15.7	3,217	16.2	3,428	15.7
Two	1,904	4.5	1,666	4.2	926	4.7	997	4.6
Three or more	1,581	3.7	1,360	3.5	737	3.7	863	4.0
Family history of breast cancer in first degree relative (male or female)								
No	29,536	83.5	27,782	83.8	13,814	83.6	14,930	83.4
Yes	5,838	16.5	5,356	16.2	2,708	16.4	2,978	16.6

^a^Missing values were excluded from percentage calculations.

**Table 2:** Distribution of select risk factors across categories of nonaspirin nonsteroidal anti-inflammatory drug (NSAID) use among 126,124 women, National Institutes of Health–AARP Study

	Frequency of nonaspirin NSAID use in past 12 months							
	
	None		<weekly		1–6 times/week		1+/day	
	
Characteristic	*n*	%^a^	*n*	%^a^	*n*	%^a^	*n*	%^a^
Age at second questionnaire								
<57 years	7,112	14.3	9,185	23.4	4,563	24.0	2,955	17.9
57–60 years	8,605	17.3	8,288	21.1	4,171	21.9	3,052	18.4
61–64 years	11,482	23.0	8,854	22.5	4,278	22.5	3,876	23.4
65–68 years	14,666	29.4	8,841	22.5	4,039	21.2	4,399	26.6
69+ years	7,962	16.0	4,136	10.5	1,962	10.3	2,263	13.7
Race/ethnicity								
Caucasian/non-Hispanic white	44,909	90.1	35,940	91.4	17,537	92.2	15,103	91.3
Other/unknown	4,918	9.9	3,364	8.6	1,476	7.8	1,442	8.7
Education								
<High school/high school grad	16,007	33.1	10,402	27.1	5,595	30.3	4,734	29.5
Post-high school+	32,364	66.9	27,959	72.9	12,851	69.7	11,312	70.5
Body mass index at baseline								
<25 kg/m^2^	23,279	48.6	18,527	48.6	7,800	42.4	5,418	33.9
25–30 kg/m^2^	15,189	31.7	12,423	32.6	6,194	33.7	5,296	33.2
30+ kg/m^2^	9,464	19.7	7,193	18.9	4,413	24.0	5,254	32.9
Smoking								
Never	22,702	47.0	17,354	45.5	8,254	44.8	7,062	44.0
Former	18,554	38.4	15,751	41.3	7,708	41.9	6,942	43.2
Current	7,025	14.6	5,023	13.2	2,453	13.3	2,050	12.8
Alcoholic drinks								
0 drinks per day	16,225	32.6	9,230	23.5	4,728	24.9	5,121	31.0
<1 drink per day	27,087	54.4	24,388	62.0	11,660	61.3	9,312	56.3
1–3 drinks per day	5,108	10.3	4,570	11.6	2,153	11.3	1,616	9.8
3+ drinks per day	1,407	2.8	1,116	2.8	472	2.5	496	3.0
Self-reported general health								
Excellent/very good/good	42,891	87.5	35,824	92.4	16,655	88.8	13,073	80.5
Fair/poor	6,142	12.5	2,966	7.6	2,106	11.2	3,176	19.5
Stroke								
No	48,762	97.9	38,882	98.9	18,760	98.7	16,223	98.1
Yes	1,065	2.1	422	1.1	253	1.3	322	1.9
Heart disease								
No	44,964	90.2	36,916	93.9	17,658	92.9	14,936	90.3
Yes	4,863	9.8	2,388	6.1	1,355	7.1	1,609	9.7
High blood pressure								
No	27,666	59.6	23,960	65.1	10,656	60.1	8,036	51.7
Yes	18,783	40.4	12,847	34.9	7,080	39.9	7,508	48.3
Mammogram in past 3 years								
Once or less	17,403	35.3	10,768	27.6	5,095	27.0	4,509	27.5
Yes, more than once	31,949	64.7	28,229	72.4	13,749	73.0	11,904	72.5
Age at menarche								
<13 years	23,714	48.1	18,717	48.0	9,436	50.0	8,816	53.7
13–14 years	21,018	42.6	16,749	43.0	7,752	41.1	6,269	38.2
15+ years	4,604	9.3	3,516	9.0	1,685	8.9	1,336	8.1
Parity								
Nulliparous	8,010	16.4	5,540	14.3	2,375	12.7	2,212	13.6
One	5,330	10.9	4,022	10.4	1,794	9.6	1,546	9.5
Two	12,441	25.5	10,535	27.2	5,125	27.3	4,095	25.2
Three or more	23,059	47.2	18,566	48.0	9,445	50.4	8,418	51.7
Age at first live birth								
Nulliparous	8,010	16.4	5,540	14.3	2,375	12.6	2,212	13.6
<20 years	7,690	15.7	6,056	15.6	3,559	19.0	3,229	19.8
20–24 years	21,059	43.0	17,194	44.4	8,585	45.7	7,350	45.1
25–29 years	9,058	18.5	7,520	19.4	3,310	17.6	2,676	16.4
30+ years	3,153	6.4	2,439	6.3	948	5.0	844	5.2
Age at menopause								
Premenopausal	1,145	2.3	1,864	4.7	886	4.7	487	2.9
<45 years	3,654	7.3	2,377	6.0	1,063	5.6	995	6.0
45–49 years	8,216	16.5	5,941	15.1	2,732	14.4	2,288	13.8
50–54 years	14,355	28.8	11,383	29.0	4,705	24.7	3,895	23.5
55+ years	3,209	6.4	2,519	6.4	1,019	5.4	945	5.7
Surgical menopause	17,713	35.5	13,938	35.5	7,962	41.9	7,430	44.9
Postmenopausal, age unknown	1,535	3.1	1,282	3.3	646	3.4	505	3.1
Hormone therapy formulation								
Never used	23,083	48.6	14,199	37.8	5,961	32.9	5,179	32.8
Estrogen therapy only	12,664	26.7	10,499	27.9	5,910	32.7	5,522	35.0
Estrogen–progestin therapy	11,762	24.8	12,876	34.3	6,224	34.4	5,072	32.2
Moderate/vigorous physical activity during past 10 years								
Never	1,922	3.9	1,004	2.6	551	2.9	749	4.6
Rarely	5,434	11.1	3,953	10.2	1,993	10.6	2,032	12.5
<1 hour/week	5,034	10.3	4,196	10.8	1,980	10.6	1,815	11.2
1–3 hours/week	12,127	24.8	10,252	26.4	4,815	25.7	4,091	25.2
4–7 hours/week	12,378	25.3	10,169	26.2	4,981	26.6	3,924	24.1
>7 hours/week	12,017	24.6	9,234	23.8	4,397	23.5	3,642	22.4
Number breast biopsies								
None	37,903	76.9	29,547	75.9	14,113	74.9	12,243	74.7
One	7,549	15.3	6,236	16.0	3,114	16.5	2,727	16.6
Two	2,042	4.1	1,779	4.6	884	4.7	768	4.7
Three or more	1,793	3.6	1,373	3.5	743	3.9	652	4.0
Family history of breast cancer in first degree relative (male or female)								
No	33,976	83.6	27,478	83.7	13,197	83.8	11,295	82.8
Yes	6,643	16.4	5,346	16.3	2,554	16.2	2,347	17.2

^a^Missing values were excluded from percentage calculations.

The 126,124 women accrued 836,863 person-years during an average follow-up of 3.43 years for cases (range, 1 day to 7.13 years) and of 6.75 years for noncases (range, 1 day to 7.17 years). The mean (standard deviation) ages for entry and exit were 63.1 (5.2) years and 66.5 (5.6) years for cases and were 62.6 (5.4) years and 69.3 (5.4) years for noncases, respectively. Breast cancer risk factors in this population were generally consistent with established associations with age, BMI, ages at menarche, first birth and menopause, parity, estrogen–progestin therapy use, number of breast biopsies, and family history of breast cancer.

Because NSAID exposure was collected on the second questionnaire, and the response rate for this questionnaire was ~60%, we also examined the association between breast cancer risk factors captured on the first questionnaire and breast cancer risk among the entire cohort. The associations between risk factors and breast cancer in the entire cohort were comparable with those observed in the subcohort of respondents to the second questionnaire (data not shown).

### Nonsteroidal anti-inflammatory drug use and breast cancer

In multivariate proportional hazards models, there was no statistically significant association between ever-use of NSAIDs in the past 12 months and total breast cancer (Table 3). Compared with nonusers, the relative risks (RRs) of breast cancer associated with ever-use of aspirin only, with nonaspirin NSAIDs only, or with both were 0.97 (95% CI = 0.88 to 1.07), 1.01 (95% CI = 0.92 to 1.12), and 0.95 (95% CI = 0.87 to 1.04), respectively. We tested associations by frequency of NSAID type. The risk of total breast cancer was slightly reduced with daily use (versus nonuse) of aspirin (RR = 0.93, 95% CI = 0.85 to 1.01), albeit statistically nonsignificantly (for trend *P *= 0.08). Compared with nonuse, daily use of nonaspirin NSAIDs was not associated with breast cancer risk (RR = 0.96, 95% CI = 0.87 to 1.05). The association between NSAID use and breast cancer risk was not modified by age, HT use, BMI, or smoking (data not shown).

**Table 3:** Association between nonsteroidal anti-inflammatory drug (NSAID) use and breast cancer among 126,124 women, National Institutes of Health–AARP Study

	Number of cancers	Person-years	Relative risk^a^	95% confidence interval	*P *value for trend
NSAID use					
Never	667	124,265	1.00 (referent)		n/a
Aspirin only	1,085	203,145	0.97	0.88 to 1.07	
Non-aspirin NSAID only	874	157,017	1.01	0.92 to 1.12	
Both	1,781	334,792	0.95	0.87 to 1.04	
Aspirin use^b^					
Never	1,556	284,342	1.00 (referent)		0.08
<1/week	1,405	265,676	0.95	0.89 to 1.03	
1–6/week	716	133,331	0.95	0.87 to 1.04	
1+/day	774	145,317	0.93	0.85 to 1.01	
Non-aspirin NSAID use^b^					
Never	1,764	329,944	1.00 (referent)		0.58
<1/week	1,415	262,000	1.00	0.93 to 1.07	
1–6/week	693	126,373	1.02	0.93 to 1.11	
1+/day	585	109,098	0.96	0.87 to 1.05	

^a^Adjusted for age (continuous), race, age at first birth, hormone therapy use, number of breast biopsies, alcohol intake, history of hypertension, and family history of breast cancer in first-degree relative.

^b^These models also include terms for frequency of use of opposite NSAID type.

Several differences emerged in analyses stratified by ER status and stage (Tables 4 to 6). The RR of ER-positive breast cancer was reduced with daily aspirin use (RR = 0.84, 95% CI = 0.71 to 0.98), and no association was observed with daily use of nonaspirin NSAIDs (RR = 0.98, 95% CI = 0.83 to 1.16) (Table 4). In contrast, ER-negative breast cancer was not associated with daily aspirin or nonaspirin NSAID use. The inverse relationships with aspirin use were weak for invasive breast cancer but were stronger for *in situ *disease (Table 5), and these differences were not entirely explained by mammographic screening: in analyses restricted to women who reported having two or more mammograms in the past three years, the risks associated with daily aspirin use for invasive and *in situ *breast cancers were 0.95 (95% CI = 0.85 to 1.06) and 0.75 (95% CI = 0.59 to 0.95), respectively (Table 6). There was little variation in risks for nonaspirin NSAIDs according to ER status and stage. Risk relationships for aspirin and nonaspirin NSAIDs did not vary by histologic subtype for invasive cancers (data not shown).

**Table 4:** Association between nonsteroidal anti-inflammatory drug (NSAID) use and breast cancer by estrogen receptor status, National Institutes of Health–AARP Study

	Estrogen receptor-positive					Estrogen receptor-negative				
	
NSAID use	Number of cancers	Person-years	Relative risk^a^	95% confidence interval	*P *value for trend	Number of cancers	Person-years	Relative risk^a^	95% confidence interval	*P *value for trend
Aspirin use^b^										
Never	493	280,706	1.00 (referent)		0.06	88	279,308	1.00 (referent)		0.54
<1/week	464	262,491	0.98	0.86 to 1.11		93	261,137	1.09	0.81 to 1.47	
1–6/week	243	131,724	1.00	0.86 to 1.17		42	131,022	1.01	0.70 to 1.47	
1+/day	223	143,476	0.84	0.71 to 0.98		52	142,852	1.14	0.81 to 1.62	
Nonaspirin NSAID use^b^										
Never	541	325,816	1.00 (referent)		0.86	108	324,250	1.00 (referent)		0.64
<1/week	466	258,767	1.04	0.92 to 1.18		96	257,460	1.08	0.81 to 1.43	
1–6/week	230	124,804	1.07	0.92 to 1.26		36	124,115	0.85	0.58 to 1.25	
1+/day	186	107,763	0.98	0.83 to 1.16		36	107,241	0.97	0.66 to 1.42	

The threshold for a positive estrogen receptor was ≥10 fmol receptor/mg total protein. ^a^Adjusted for age (continuous), race, age at first birth, hormone therapy use, number of breast biopsies, alcohol intake, history of hypertension, and family history of breast cancer in first-degree relative. ^b^These models also include terms for frequency of use of opposite NSAID type.

**Table 5:** Association between nonsteroidal anti-inflammatory drug (NSAID) use and breast cancer according to stage, National Institutes of Health–AARP Study

	*In situ *breast cancer					Invasive breast cancer				
	
NSAID use	Number of cancers	Person-years	Relative risk^a^	95% confidence interval	*P *value for trend	Number of cancers	Person-years	Relative risk^a^	95% confidence interval	*P *value for trend
Aspirin use^b^										
Never	298	280,077	1.00 (referent)		0.02	1,254	283,247	1.00 (referent)		0.37
<1/week	233	261,641	0.80	0.67 to 0.96		1,166	264,821	0.99	0.91 to 1.07	
1–6/week	124	131,305	0.85	0.69 to 1.05		589	132,899	0.98	0.88 to 1.08	
1+/day	122	143,137	0.78	0.63 to 0.96		648	144,854	0.96	0.87 to 1.06	
Non-aspirin NSAID use^b^										
Never	292	324,890	1.00 (referent)		0.45	1,463	328,884	1.00 (referent)		0.88
<1/week	269	258,097	1.14	0.96 to 1.35		1,140	261,013	0.97	0.90 to 1.05	
1–6/week	122	124,433	1.06	0.85 to 1.31		569	125,934	1.01	0.92 to 1.12	
1+/day	91	107,466	0.87	0.69 to 1.10		494	108,757	0.98	0.89 to 1.09	

^a^Adjusted for age (continuous), race, age at first birth, hormone therapy use, number of breast biopsies, alcohol intake, history of hypertension, and family history of breast cancer in first-degree relative.

^b^These models also include terms for frequency of use of opposite NSAID type.

**Table 6:** Association between nonsteroidal anti-inflammatory drug (NSAID) use and breast cancer according to stage among screened women, National Institutes of Health–AARP Study

	*In situ *breast cancer					Invasive breast cancer				
	
NSAID use	Number of cancers	Person-years	Relative risk^a^	95% confidence interval	*P *value for trend	Number of cancers	Person-years	Relative risk^a^	95% confidence interval	*P *value for trend
Aspirin use^b^										
Never	241	192,125	1.00 (referent)		0.02	916	194,396	1.00 (referent)		0.15
<1/week	180	179,747	0.77	0.63 to 0.93		847	181,986	0.98	0.89 to 1.07	
1–6/week	97	90,680	0.82	0.65 to 1.05		402	91,715	0.90	0.80 to 1.01	
1+/day	96	99,793	0.75	0.59 to 0.95		476	101,064	0.95	0.85 to 1.06	
Non-aspirin NSAID use^b^										
Never	218	209,044	1.00 (referent)		0.50	982	211,655	1.00 (referent)		0.62
<1/week	222	185,624	1.16	0.95 to 1.40		852	187,741	0.98	0.89 to 1.07	
1–6/week	97	90,035	1.03	0.81 to 1.31		428	91,161	1.03	0.92 to 1.16	
1+/day	76	77,462	0.89	0.69 to 1.16		384	78,439	1.02	0.91 to 1.15	

This analysis was restricted to women who reported ≥2 mammograms in the past 3 years. ^a^Adjusted for age (continuous), race, age at first birth, hormone therapy use, number of breast biopsies, alcohol intake, history of hypertension, and family history of breast cancer in first-degree relative. ^b^These models also include terms for frequency of use of opposite NSAID type.

## Discussion

In the present large prospective study, we found that NSAID use was unrelated to risk of total breast cancer. Daily aspirin, but not nonaspirin NSAID, use was associated with a modest 16% reduction in ER-positive breast cancer. We did not observe an association between NSAID use and ER-negative breast cancer.

Our null findings for total breast cancer are consistent with those reported by eight prospective studies [8-15] and one randomized clinical trial [25] that examined aspirin and/or nonaspirin NSAID use. Our results differ, however, from those from seven prospective studies [17,19-24] and 12 case–control studies [1-3,30-33,40-44], most of which have reported reduced risks of breast cancer associated with NSAID use. The reasons for these differing results are unclear, but associations may be limited to certain subtypes of breast cancer. Differences in NSAID exposure assessment across studies may also account for inconsistent results.

The frequency of aspirin and nonaspirin use in the NIH–AARP Diet and Health Study is consistent with that reported in other US cohorts of women [21,45], and the proportion of women in this study using any NSAID is only slightly higher than that reported among females ages 45 to 75+ years in the third National Health and Nutrition Examination Survey – 71.5% to 80.4% of whom reported any nonprescription analgesic in the past month [46]. Our study lacked information, however, on the duration, dose, indication, or prescription NSAID use. Because we only measured recent NSAID use in the year prior to the 1996 to 1997 questionnaire and followed women through 2003, the null findings we observed for NSAIDs may differ from what might be observed with long-term cumulative exposure. Indeed, several cohort studies have shown that long-term NSAID use is required for an observed chemopreventive effect on breast cancer [22,23]. Furthermore, if there were individuals in our referent category who were past users or prescription NSAID users, results could be biased toward the null.

In addition, we were unable to separate low-dose from regular-dose aspirin use; results from the Women's Health Study randomized trial [25], a study conducted using an insurance database [2], and laboratory evidence [27] suggest that doses higher than the 80 mg aspirin per day typically recommended for cardiovascular therapy may be required to permanently inactivate COX-2. If low-dose aspirin users were included in our exposed group, thereby diluting any effect associated with regular-dose aspirin use, the results would be biased toward the null. Although we did not collect information on indication for use, it is likely that some daily users were taking aspirin for heart disease prevention given that daily users more frequently reported history of heart disease. To address the concern that women using NSAIDS for cardioprotection may be under closer medical supervision and/or may be more health conscious, we additionally controlled for self-reported heart disease, self-rated health quality, and mammographic screening – and the results remained unchanged.

Despite these limitations, the NIH–AARP Diet and Health Study is the one of largest cohorts to date to have evaluated the association between NSAID type and breast cancer risk by tumor characteristics, including ER status. The reduced risk of ER-positive breast cancer we observed with daily aspirin use is consistent with the mechanism of action of aspirin (but not nonaspirin NSAIDs), which permanently inactivates COX-2 [29], potentially reducing breast cancer risk via multiple pathways, including suppression of estrogen synthesis by decreased aromatase activity [28]. In addition, our results are in agreement with findings from the Long Island Breast Cancer Prevention Project, a population-based case–control study that reported in 2004 an inverse association with aspirin that was significantly greater for HR-positive breast cancer than for HR-negative breast cancer [30]. Since then, two case–control studies [3,31] and three prospective cohort studies [16,17,24] have separately investigated the association for aspirin, with three of the five studies suggesting some protection against HR-positive breast cancer with aspirin use [3,16,31].

A US hospital-based study – the Case–Control Surveillance Study – reported a 26% nonsignificant reduction (95% CI = 0.44 to 1.26) in HR-positive breast cancer associated with regular (versus <4 times/week) aspirin use [31]. More recently, a Canadian population-based case–control study found a 31% reduction (95% CI = 0.56 to 0.86) in ER-positive/PR-positive breast cancer associated with daily (versus <daily) aspirin use [3]. The California Teachers Study followed 114,460 women aged 22 to 85 years for 6 years and found that long-term daily (versus <once/week) aspirin use was associated with a statistically nonsignificant decreased risk of ER-positive/PR-positive breast cancer (RR = 0.80, 95% CI = 0.62 to 1.03) [16]. In contrast, the Multiethnic Cohort Study and the Danish Diet, Cancer and Health Cohort Study found no protective effect of aspirin use for HR-positive or HR-negative breast cancer [17,24]. Finally, the Women's Health Study randomized clinical trial did not observe any significant patterns of risk by HR status with low-dose aspirin use [25]. Therefore, while our findings, along with several others, suggest at least some reduction in ER-positive breast cancer associated with aspirin use, the evidence is not conclusive and additional prospective studies with detailed exposure data on the dose, frequency, duration, and indication are needed.

We found stronger inverse associations with *in situ *breast cancer than invasive breast cancer. Among the few prior studies that have reported results separately for *in situ *breast cancer and invasive breast cancer, two case–control studies reported decreased risks of both *in situ *and invasive breast cancers associated with aspirin [30] and with any NSAID use [32], but the findings for *in situ *breast cancer were not statistically significant in either study. The Iowa Women's Health cohort study reported a reduction in breast cancer risk with increased frequency of aspirin, but not with nonaspirin NSAIDs, for both *in situ *and invasive disease [21]. Both *in situ *and invasive breast tumors express COX-2 [47], suggesting that upregulation of COX-2 may be an early event in carcinogenesis. Higher rates of COX-2 expression in *in situ *compared with invasive tumors have led investigators to suggest that the potential therapeutic impact of COX-2 inhibition may be more relevant for *in situ *breast cancer than invasive breast cancer (reviewed in [48]). The inverse association for *in situ *breast cancer associated with aspirin use in the present study may therefore be biologically plausible and warrants further investigation.

We observed no variation in risk with HT, smoking status, or BMI, and our findings are generally consistent with previous investigations. For the most part, tests for interactions between HT and NSAID use have been statistically nonsignificant across case–control studies [30,33] and cohort studies [13,17,22,24,25]; however, two cohort studies have suggested the protective effect of NSAIDs may be attenuated among HT users [18,20]. In line with data suggesting that proinflammatory tobacco carcinogens may alter the effectiveness of chemopreventive agents [49], the Women's Health Study found that low-dose aspirin use was protective among former smokers but was associated with an increased risk among never smokers (for interaction *P *= 0.09) [25]. We, along with a Canadian population-based case–control study, however, did not observe effect modification by smoking [3]. Consistent with our findings, two case–control studies [32,33] and seven prospective studies [13,17,18,20,22,24,25] have reported no significant interactions between BMI and NSAID use with respect to breast cancer risk.

## Conclusion

In summary, our results do not support an important influence of NSAIDs on total breast cancer risk. Daily aspirin use, however, appeared to offer some protection for ER-positive breast cancer in this population. In addition, our findings suggest that the associations for aspirin use may vary by tumor stage. Our results provide support for further evaluating relationships in prospective studies with well-defined measures of NSAID use by NSAID type, by breast cancer stage, and by ER status.

## Abbreviations

BMI = body mass index; CI = confidence interval; COX = cyclooxygenase; ER = estrogen receptor; HR = hormone receptor; HT = hormone therapy; NIH = National Institutes of Health; NSAID = nonsteroidal anti-inflammatory drug; RR = relative risk.

## Competing interests

The authors declare that they have no competing interests.

## Authors' contributions

AS, ARH, and MFL participated in the acquisition of data. GLG, LAB, JVL Jr, AS, and MFL were involved with the study concept and design. GLG, JVL Jr, MFL, DR, and LAB contributed to the statistical analyses. GLG, JVL Jr, and LAB participated in manuscript preparation. All authors participated in the interpretation of results and critical revision of the manuscript for important intellectual content. All authors read and approved the final manuscript.
